# Downregulation of Long Noncoding RNA MIAT in the Retina of Diabetic Rats with Tail-vein Injection of Human Umbilical-cord Mesenchymal Stem Cells

**DOI:** 10.7150/ijms.38078

**Published:** 2020-02-17

**Authors:** Chuan Yu, Kun Yang, Xuxia Meng, Bowen Cao, Fenglei Wang

**Affiliations:** 1 Ophthalmology, Affiliated Hospital of Qingdao University, Qingdao 266500, Shandong Province, China.; 2 Central Laboratory, Affiliated Hospital of Qingdao University, Qingdao 266500, Shandong Province, China.; 3 Center for Ophthalmology, Institute for Ophthalmic Research, University of Tuebingen, 72076, Tuebingen, Germany.

**Keywords:** Diabetic retinopathy, Human umbilical-cord mesenchymal stem cells, Long noncoding RNA MIAT, Microangiopathy, Inflammation

## Abstract

Diabetic retinopathy (DR) is the common and important cause for visual impairment and blindness in working-aged people. Microangiopathy and inflammatory reactions are the key components of DR. Recently, long non-coding RNA myocardial infarction-associated transcript (MIAT) has emerged as a vital player in regulation for inflammatory processes and microvascular dysfunction. Additionally, cell-based therapy provides a potential option for the treatment of DR. The anti‐inflammatory effects and repair therapy of mesenchymal stem cells (MSCs) have been paid more attention. This study investigated the effects of human umbilical-cord mesenchymal stem cells (HUMSCs) injection on diabetic rat model. The results show that the level of MIAT is significantly decreased in the diabetic retina after the injection of HUMSCs. Moreover, HUMSCs can significantly decrease the expression of IL-1β and IL-6 mRNA; alleviate microvascular permeability, and upregulate Occludin expression. Studies have shown that MIAT knockdown could alleviate diabetes-induced inflammation responses and vascular leakage. Furthermore, our findings also showed that the expression of MIAT was positively correlated with the expression of IL-1β and IL-6. These results suggest that MIAT might play important regulatory roles in alleviating inflammatory reactions and microangiopathy inducing by DR after transplantation of HUMSCs.

## Introduction

As the most frequent and serious microvascular complication of diabetes mellitus (DM), Diabetic retinopathy (DR) is the leading cause of blindness among working‐age adults in many developed countries, and worldwide, the number of people with impairment or loss of vision resulting from DR is gradually increasing 1-4. The early pathogenesis of DR is mainly due to chronic damage of retinal vascular tissue, including microaneurysm, vascular leakage and capillary occlusion 5. The mechanisms of microvascular abnormalities include tight junction disassembly 6, endothelial cell-mediated leukostasis 7, capillary basement membrane thickening and pericyte or vascular smooth muscle cell detachment 5, which result in breakdown of blood-retinal barrier (BRB), an important early sign of DR 6. Moreover, BRB breakdown conduce to the increase of vascular permeability in DR 6.

Previous researches have shown that DR has different manifestations of abnormality, not only limited to microangiopathy 8,9. Inflammatory processes also play a conspicuous role in the pathogenesis of DR, especially at the early stage of DR 10,11. In diabetic retina, multiple inflammatory mediators are known to be upregulated, inducing inflammatory responses that cause disruption of tight junction and endothelial apoptosis 8. Increased proinflammatory cytokines such as interleukin-1β (IL-1β) and interleukin-6 (IL-6), promote BRB breakdown and increase microvascular impairment, which contribute to the increase of vascular permeability by different signaling pathways in diabetes-induced retinal pathology 12-14. However, studies have verified that inhibition of inflammatory processes can block the development of DR 10,15.

Mesenchymal stem cells (MSCs) are embryonic mesoderm-derived adult stem cells which have self‐renewal capacity and differential potential to a wide variety of cell types 16. Studies have showed that the damaged tissues repair and anti‐inflammatory effects of MSCs have been paid more attention. In wound repair model, human gingiva-derived mesenchymal stem cells could promote M2 polarization of macrophages to accelerate wound healing 17. Similarly, human umbilical-cord MSCs (HUMSCs) has also been demonstrated to direct macrophages into an anti‐inflammatory phenotype to mitigate insulin resistance partly 18. Sun and colleagues reported that HUMSCs could decrease inflammatory cytokines by suppressing NLRP3 inflammasome activation in type 2 diabetes rats 19. Moreover, HUMSCs are currently under clinical research to evaluate the safety and efficacy of treatment of type 2 diabetes 20. However, the roles of HUMSCs in DR have not been elucidated, and to probe the potential mechanisms is indispensable for the clinical application.

Long noncoding RNAs (LncRNAs) are usually described to be a family of RNAs transcripts with the length of more than 200 nucleotides and limited protein-coding capacity. These noncoding functional RNAs are shown as regulators to execute diverse molecular functions in multiple biological processes, such as development, differentiation, and metabolism 21. Myocardial infarction-associated transcript (MIAT), previously known as RNCR2 (retinal non-coding RNA2) and GOMAFU, was first identified as lncRNA in the study of susceptibility of myocardial infarction 22. A study has demonstrated MIAT is a critical regulator of vascular integrity and neuronal function in the eye and brain 23. The abnormal expression of MIAT was implicated in various pathological processes, such as myocardial infarction 22, microvascular dysfunction 24 and DR 25. In addition, researchers revealed that MIAT plays an important regulatory role in inflammation-related processes. MIAT knockdown can't only reverse the inflammation-induced negative effects to promote osteogenesis of human adipose-derived stem cells 26, but also inhibit the upregulation of diabetes mellitus-induced proinflammatory factors to alleviate retinal inflammation 24. Li et al 27 have reported that LncRNA MALAT1 could promotes immunosuppressive properties of MSCs, and MALAT1-overexpressed MSCs promoted M2 macrophage polarization by MALAT1-induced IDO expression to enhance the immunosuppressive properties of MSCs in vivo. However, whether MIAT has a regulative effect on treatment of DR after HUMSCs injection is still unclear.

To identify the mechanisms of MIAT in treating DR by HUMSCs, we studied the effects of HUMSCs on the expression of MIAT, proinflammatory cytokines and the microangiopathy in diabetic retina after tail-vein injection of HUMSCs by using STZ-induced diabetic rat models; and the correlation between MIAT and proinflammatory cytokines.

## Materials and methods

### Induction and grouping of Model

The study was approved by the Qingdao Animal Ethical Committee and conducted according to the guidelines of the ARVO Statement for the use of animals in ophthalmic and vision research. Male Sprague-Dawley rats (6-8 weeks old, 180~210g) were injected intraperitoneally with a single dose of STZ (60mg/kg) for the induction of diabetes. The rats with blood glucose level (≥16.7mmol/L) were included in the study and considered as the DM group. The control group (n=15) received the same dosage of sterile saline. Rats were housed under the 12h light-dark cycle and fed on chow and water freely. The diabetic rats continued to receive feed on a high sugar and fat diet until the end of the study. The care procedures that all animal received were performed according to the criteria outlined in the institutional and National Institute of Health guidelines.

The rats of the DM group were randomly divided into different groups: DM 3 months (DM 3m) group (n=15), 3 months after building of diabetes model; DM 4m group (n=15); DM 5m group (n=15); DM 6m group (n=15); DM+HUMSCs group (n=15), transplanted with HUMSCs at 5 months after DM induction; DM+PBS group (n=15), injected with phosphate buffer solution (PBS) at 5 months after DM induction. Rats in DM+HUMSCs group and DM+PBS group were treated respectively with tail-vein infusion of HUMSCs (1ml, cell number: 5×10^6^/rat) and PBS (1ml) at the fifth month after diagnosed as diabetic rats. After 1 month of injection, the rats were sacrificed.

### Preparation of HUMSCs

Human umbilical cord collection was approved by the Ethics Committee of the Affiliated Hospital of Qingdao University. The umbilical cords were obtained from fully consenting patients, and then collected in sterile phosphate buffer saline (PBS) at 4 °C. The isolation and identification of HUMSCs followed the methods previously described 28, with some modifications. The umbilical cords were washed twice with PBS, the vessels being removed at the same time. Subsequently, the rest was dissected into smaller fragments (approximately 1-2 cm^3^ in volume). These fragments were transferred to a culture-flask in low glucose-DMEM medium supplemented with 10% fetal bovine serum, penicillin and streptomycin. The cord fragments were hatched for 72h in moist environment with 5% CO^2^ at 37 °C. The tissue and non-adherent cells were washed thereafter. The medium was renewed twice a week. When the fusion degree had reached 80% or more, the adherent cells were separated with trypsin digestion and passaged in aseptic culture dish. Afterward, these mesenchymal cells were cultured for further expansion to 4 passages. The final cells were examined to be qualified by double staining, flow cytometry and genetics. These cells had typical morphological characteristics, specific immunophenotype (positive expression of CD73 and CD90 but not CD34, CD45 and HLA-DR) and normal chromosomal karyotype.

### Quantitative RT-PCR

Quantitative RT-PCR was used to detect LncRNA MIAT, IL-6 mRNA and IL-1β mRNA in rat retina. Total RNA was extracted from the retinal specimens using Trizol reagent (Invitrogen, US), and genomic DNA in the extract was eliminated by DNase (Qiagen). cDNA was synthesized with reverse transcription with the Prime Script TM RT reagent Kit (Takara). The sequences of the primers were: CTTCGAGTACAAAAACGCGTCAC (sense) and TCAGATTGCTCGCCAGTTCC (antisense) for MIAT; CAGCGATGATGCACTGTCAGA (sense) and TCCAGAAGACCAGAGCAGATTTTC (antisense) for IL-6; GCTTCAAATCTCACAGCAGCATC (sense) and CGTCATCATCCCACGAGTCAC (antisense) for IL-1β; TATCGGACGCCTGGTTACCA (sense) and ACTGTGCCGTTGAACTTGCC (antisense) for GAPDH. Every 20 μl reaction system contained 10 μl SYBR green mix, 6.4μl nuclease-free water, 2 μl cDNA, 0.8μl forward primers and 0.8 μl reverse primers.

GAPDH was used as the internal standard. The results were represented with the threshold cycle (CT) values. The expression of detected gene was standardized to GAPDH and analyzed using the 2^-ΔΔCT.

### Retinal Evans Blue Leakage Assay

Retinal Evans blue (EB) leakage assay was carried out as described by Copland et al 29. EB (20 mg/mL in saline, Sigma, US) was injected at a dose of 45 mg/kg through internal jugular vein. Rats were sacrificed after 2 hours circulation. The eyes were picked out and immersed into 4% paraformaldehyde for 1 hour. EB vascular leakage was observed by fluorescence microscope. EB leakage amount was calculated in the fellow eye. Rats were perfused via the left ventricle after the dyestuff had circulated for 2 hours. Both eyes were enucleated as soon as the perfusion was finished, then to separate and dry the retinas. In order to extract the dyestuff, the retinas were incubated in 0.3ml formamide for 18 hours at 70°C. The concentration of EB in the extracts was analyzed by absorbance differences and quantified according to previously described 30.

### Immunohistochemistry

Four-micrometer serial paraffin sections were made after eyes were fixed in 4% paraformaldehyde. Then the sections were deparaffined and washed routinely, and incubated with 3% hydrogen peroxide to block the nonspecific process. Antigen repair was employed in the sodium citrate buffer. Retinal sections were washed and blocked with normal goat serum fluid, and then incubated respectively with the primary antibody, polyclonal rabbit anti-rat Occludin (Elab science); and the secondary antibody, goat anti-rabbit IgG. The specimens were incubated with the solution of horseradish labeled streptavidin. Immunoreactivity was observed with a diaminobenzidine substrate kit (Solar bio). Images were obtained with light microscope.

The positive area and the optical density (OD) values of the immunostaining were measured using Image Pro Plus 6.0.

### Statistical Analysis

All statistical analysis was performed with the IBM SPSS 22.0 software. Results were shown as the mean ± standard deviation (SD). The statistical significance of between-group differences were tested by one-way ANOVA and followed by the least significant difference (LSD) test. The correlation among data was analyzed using the regression analysis. P < 0.05 was considered statistically significant.

## Results

### MIAT levels of different months in the retinas of diabetic rats

**Figure 1:**
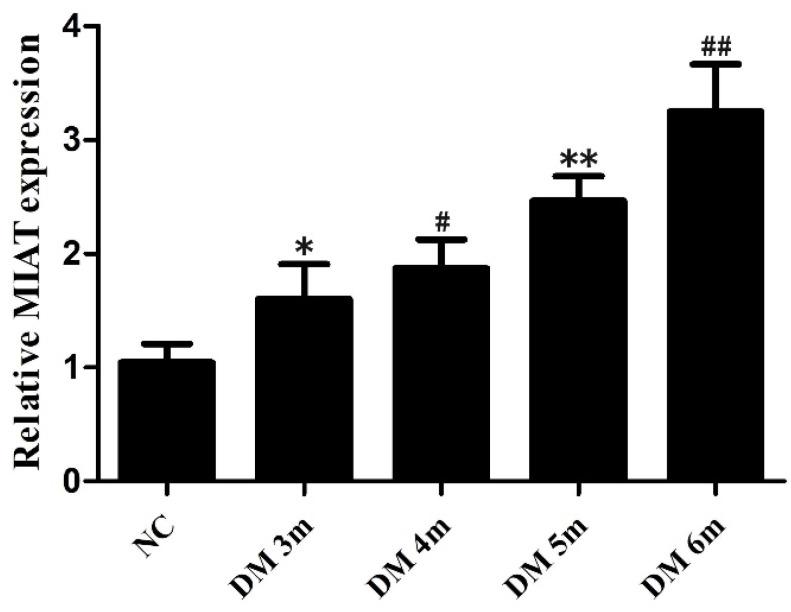
MIAT expression levels of different months in the retinas of diabetic rats. The adjacent months *P>0.05 versus NG group,***^ #^**** P*>0.05 versus DM 3m group, **P>0.05 versus DM 4m group, ***^##^****P*>0.05 versus DM 5m group; the non-adjacent months ***^#^****P*< 0.05, **P< 0.05,***^##^**P*< 0.05 versus NG group, **P < 0.05 versus DM 3m group, ***^##^****P* < 0.05 versus DM 3m group, ***^##^****P* < 0.05 versus DM 4m group.

The expression of MIAT was obviously increased in the retinas of diabetic rats compared with the non-diabetic rats 24. To further determine the association between the expression of MIAT and DR progression, the total RNAs were extracted from the retinas of diabetic rats in different months. The MIAT level was increased in DM 3m group compared with the NC group, but differences were not statistically significant. The expressions of MIAT were significantly increased in DM 4m group, DM 5m group and DM 6m group compared with the NC group. Furthermore, the expression of MIAT in the adjacent months had no significant difference, while there were statistically significant in MIAT expressions between non-adjacent months. (Fig. 1) Those results suggest that high glucose conditions significantly upregulate of MIAT expression in a time-dependent manner in the retinas of diabetic rats.

### MIAT, IL-6 mRNA and IL-1β mRNA levels are significantly downregulated in the diabetic rats with injection of HUMSCs

**Figure 2:**
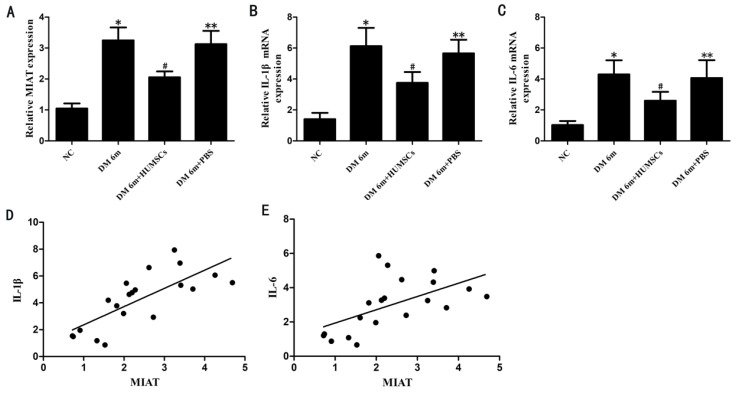
MIAT, IL-1β mRNA and IL-6 mRNA expression levels in the retinas of diabetic rats with injection of HUMSCs. (A, B, C) MIAT, IL-1β mRNA and IL-6 mRNA expression levels in different groups; (D,E) the correlation between MIAT and IL-1β, IL-6. *P< 0.05 versus NG group, ***^#^****P*< 0.05 versus NG group, ***^#^****P*< 0.05 versus DM 6m group and DM 6m+PBS group, **P> 0.05 versus DM 6m group. r_(D,E)_ =0.750, 0.537; *P=*0.000, 0.008, respectively.

To investigate the expression of MIAT, IL-1β and IL-6 in the retinas of diabetic rats with tail vein injection of HUMSCs, MIAT, IL-1β mRNA and IL-6 mRNA levels were quantified by quantitative RT-PCR. The results indicated that the expression of MIAT was significantly decreased in DM 6m+HUMSCs group compared with DM 6m group (Fig.2A). Similarly, IL-1β mRNA and IL-6 mRNA levels were also significantly downregulated in the HUMSCs injected group (Fig. 2B, C). In addition, we further investigated whether MIAT was correlated with IL-1β and IL-6. The results indicated that the expression of MIAT was positively correlated with that of IL-1β and IL-6 (Fig. 2D, E).

### HUMSCs reduced retinal microvascular permeability in diabetic rats

**Figure 3:**
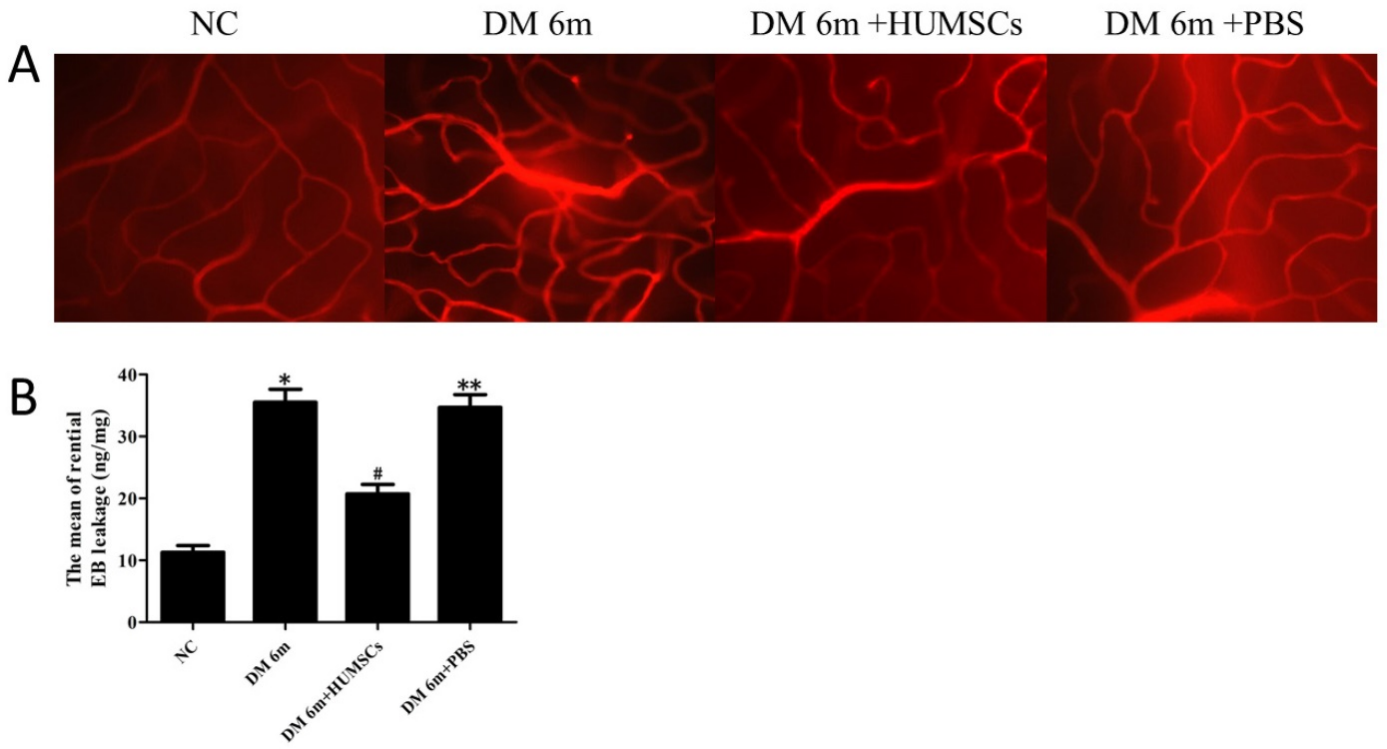
HUMSCs reduced retinal microvascular permeability in diabetic rats. (A) Microvascular permeability was evaluated by EB staining in NC group, DM 6m group, DM+HUMSCs group, DM+PBS group. Compared with the NC group, the DM 6m group and the DM+PBS group had an obvious EB leakage, whereas the leakage of EB was significantly decreased in DM+HUMSCs group. (B) Quantification of the average EB leakage. The amount of retinal EB leakage was markedly higher in DM 6m group and DM+PBS group than that in NC group, and HUMSCs could reduce the retinal vascular leakage. *P< 0.05, **P< 0.05 versus NC group;***^ #^****P*< 0.05 versus DM 6m group, DM+PBS group.

To assess the microvascular permeability transition in retinas of diabetic rats with injection of HUMSCs, retinas were handled by EB staining. The results indicated that the retinal microvascular was normally routed in the NC group with no leakage; whereas retinal microvascular leakage was observed in the DM 6m group (Fig. 3A). After 1 mouth following tail vein injection of HUMSCs, EB permeation was significantly decreased in the DM 6m+ HUMSCs group (Fig. 3A). The retinal EB leakage in DM 6m group (35.52 ± 2.11 ng/mg) evidently increased in comparison with the NC group (11.27 ± 1.12 ng/mg). Furthermore, after injection of HUMSCs, the EB leakage was distinctly reduced (20.73 ± 1.53 ng/mg). The leakage in DM 6m+PBS group (34.68 ± 2.06 ng/mg) has no difference comparing with DM 6m group (Fig. 3B).

### HUMSCs upregulate the expression of Occludin in the retina of diabetic rats

**Figure 4:**
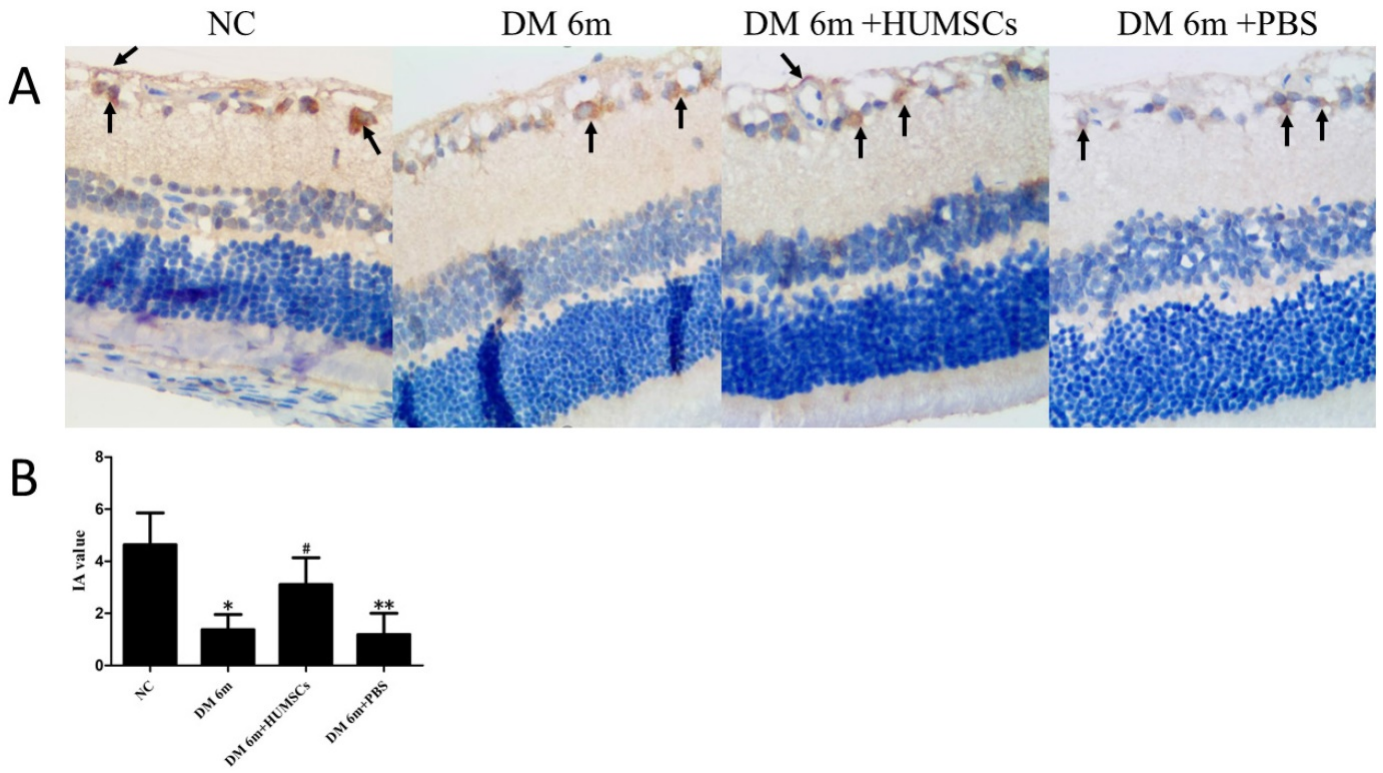
HUMSCs upregulate the expression of Occludin in the retina of diabetic rats. (A) Occludin localization in different groups. Brown granules were found in the microvascular wall of the retinal ganglion cell layer, and there were brown granules in the nerve fiber layer and the inner nuclear layer. The DM 6m+ HUMSCs group showed the higher expression of Occludin than the DR 6m group and the DM 6m+PBS group. (B) The IA value of Occludin in different groups. The IA value was distinctly lower in DM 6m group and DM+PBS group than that in NC group, while the IA value was significantly increased in DM 6m+ HUMSCs group compared with the DR 6m group and DM 6m+PBS group. *P < 0.05, **P < 0.05 versus NC group;***^ #^****P* < 0.05 versus DM 6m group, DM+PBS group.

In the immunohistochemical assay, we detected the expression of Occludin, a tight-junction protein located in endothelial cells junctions. The results indicated that the DM 6m group showed a lower expression obviously of Occludin than the NC group, while there was staining deeply in DM 6m+ HUMSCs group compared with the DR 6m group and the DM 6m+PBS group (Fig. 4A). In line with the dyeing, the mean integral absorbance (IA) value of the HUMSCs injected group (3.11 ± 1.03) was significantly increased compared with the DR 6m group (1.38 ± 0.58) and DM 6m+PBS group (1.19 ± 0.81), and the NC group (4.63 ± 1.22) showed a higher IA value than other groups (Fig. 4B).

## Discussion

Diabetic retinopathy is a blinding disease, many mechanisms of which have been put forward to interpret its pathogenesis. Currently, it has been confirmed that microvascular changes are the major characteristics in retinopathy as diabetes progresses. However, increasing evidence indicates that inflammation also plays a vital role in the progression of DR. Apart from vascular endothelial growth factor (VEGF) and tumor necrosis factor-α (TNF-α), several reports have indicated that levels of IL-1β and IL-6 are increased in the vitreous of patients with proliferative DR 31,32 and in the retina and retinal vessels of diabetic rats 33. And as important proinflammatory mediators, IL-1β and IL-6 are proved to be important pathogenic factors of microvascular complications in DR 12,14. Moreover, in the research of the mechanism of DR, Klaassen and coworkers present the evidence that inflammatory factors lead to defects of the integrity of BRB and further contribute to vascular permeability 34.

Previous studies have verified that inhibiting the inflammatory pathway can impede the development of retinal vascular abnormalities in DR 10,15,35,36.

Present anti-inflammatory therapies for DR target VEGF, chemokines, angiopoietin 2 and proteinases 37. Interestingly, cell-based therapy has been found to attenuate inflammatory response and promote repairing of tissue injury 38. Recently, accumulating evidence has strongly implied that MSCs therapy provides a good potential option for the treatment of different inflammatory diseases due to the anti-inflammatory effect of MSCs. Studies have indicated that the level of proinflammatory cytokines, IL-1β and IL-6, descended significantly in inflammatory bowel disease 39, acute pancreatitis 40 and type 2 diabetes 17 after injection of HUMSCs. Furthermore, the mechanisms for the anti-inflammatory effect of HUMSCs in inflammatory bowel disease and type 2 diabetes were through the modulation of 15-LOX-1 in macrophages 39 and suppressing NLRP3 inflammasome 17 respectively. However, the precise roles of HUMSCs in regulating the inflammatory-related progress of DR remain unexplored. This study suggested the level of MIAT decreased significantly in diabetic retinas after HUMSCs injection.

As previously mentioned, MIAT has been reported to play important roles in the retinal inflammatory reactions induced by diabetes mellitus 24. Study has showed that MIAT expression is upregulated in a time-dependent fashion in endothelial cells upon high glucose stress, and MIAT is conspicuously upregulated in the retinas of diabetic rats and patients 24. Our findings further verified that hyperglycemia triggers upregulation of MIAT expression in a time-dependent manner in the retinas of diabetic rats. In addition, the level of MIAT was significantly decreased in the diabetic retina after the injection of HUMSCs, suggesting HUMSCs could attenuate the expression of MIAT in DR. Yan et al 24 have shown that the downregulation of MIAT expression caused by MIAT shRNA injection could partly decreased the expression levels of VEGF, TNF-α, and intercellular adhesion molecule-1 (ICAM-1) in diabetic retinas, which indicated MIAT knockdown could alleviate retinal inflammation induced by hyperglycemia. Consistent with several previous studies 39,40, we found that the expression levels of IL-1β and IL-6 mRNA significantly decreased in the diabetic retinas after HUMSCs injection. Moreover, HUMSCs downregulated the expression of MIAT in DR, and the expression of MIAT was positively correlated with the expression of IL-1β and IL-6. Taken together, these results are the first to indicate that MIAT might have regulatory effects on the downregulation of IL-1β and IL-6 after HUMSC injection in DR, which implies there are potential mechanisms of MIAT mediating anti-inflammatory processes of HUMSCs.

Additionally, studies have demonstrated the increase of retinal vascular permeability in diabetes attribute to the breakdown of BRB 10,13. Bamforth et al. 41 have verified that intravitreal injection of IL-1β causes breakdown of the vascular BRB by inducing the retinal inflammatory response. Moreover, IL-1β cause the damage of the integrity of BRB in DR by apoptosis of retinal endothelial cells and leukostasis 12,42. Unlike IL-1β, IL-6 indirectly cause the increase of retinal vascular permeability through inducing the expression of VEGF 43, which is known to be a key molecule leading to BRB breakdown in diabetes 44. Studies have demonstrated HUMSCs could decreased the level of IL-1β and IL-6 in different inflammatory diseases 17,39,40. In this study, we found that HUMSCs obviously downregulated the expression of IL-1β and IL-6 mRNA, and retinal microvascular permeability is alleviated effectually. Along with all the others, these findings suggest HUMSCs might reduce microvascular permeability via suppressing inflammatory factors. Yan et al 24 have shown that MIAT knockdown reduces diabetes-induced retinal vascular leakage. Similarly, we found HUMSCs significantly decreased MIAT expression, and microvascular leakage is reduced effectively. Furthermore, the expression of MIAT was positively correlated with the expression of IL-1β and IL-6. Therefore, these findings provide further evidence to approve that MIAT plays regulatory roles in counteracting inflammatory progresses of HUMSCs in DR, and MIAT potentially mitigates microvascular permeability by downregulating the expression of proinflammatory mediators in DR.

Increased vascular permeability is tightly associated with the reduced tight junction proteins in diabetic retinas 45. Occludin is indispensable to tight junction integrity in endothelial cells, and is the main constituents of the vascular permeability barrier 46. In the present study, HUMSCs significantly upregulated the expression of Occludin and reduces microvascular permeability. Researches have testified that VEGF regulates retinal vascular permeability through altering the expression, phosphorylation and ubiquitination of Occludin 47,48. Moreover, IL-6 has been proved to induce the expression of VEGF in increasing vascular permeability 43. In this study, we found that HUMSCs could decrease the expression levels of IL-6 mRNA. The results indicated that HUMSCs might induce VEGF expression through IL-6 to regulate the level of Occludin and then cause the alteration of vascular permeability. Yan et al 24 have showed that MIAT knockdown partially downregulates VEGF expression induced by diabetes. Interestingly, MIAT regulates the function of retinal endothelial cells through MIAT/miR-150-5p/VEGF regulatory network 24. Due to the possible regulatory roles of MIAT in anti-inflammatory of HUMSCs and the correlation between MIAT and IL-6, we speculate that MIAT might have regulatory effects on the changes of Occludin expression after HUMSCs injection. However, the precise roles and underlying mechanisms have remained to be explored. Further detailed researches need to be performed to confirm the molecular mechanisms involved.

## Conclusions

Taken together, our study showed that the expression of MIAT is significantly decreased in the diabetic retina after the injection of HUMSCs. Moreover, inflammatory responses and microvascular permeability were ameliorated, and the Occludin expression was upregulated. Furthermore, MIAT might play regulatory roles in those processes. Those findings of this study provided a new insight into the mechanism of therapy of DR by HUMSCs. Whether the therapeutic effects of HUMSC depend on the stem cells implanted in retina or the nutritional factors secreted by HUMSCs will be probed. And further studies are indispensable to elucidate the potential roles and specific regulation mechanisms of MIAT in the process of HUMSCs treatment for DR.
